# Feasibility of a community-adapted multi-domain intervention for dementia prevention among older adults: a research protocol

**DOI:** 10.1186/s13690-023-01205-0

**Published:** 2023-10-31

**Authors:** Yujiro Kuroda, Kosuke Fujita, Taiki Sugimoto, Kazuaki Uchida, Taichi Shimazu, Junko Saito, Hidenori Arai, Takashi Sakurai

**Affiliations:** 1 Department of Prevention and Care Science, Center for Development of Advanced Medicine for Dementia, National Center for Geriatrics and Gerontology, 7-430 Morioka, Obu, 474-8511 Aichi, Japan; 2 Division of Behavioural Sciences, National Cancer Center Institute for Cancer Control, National Cancer Center, Tokyo, Japan; 3 National Center for Geriatrics and Gerontology, Obu, Aichi, Japan

**Keywords:** Dementia prevention, Community settings, Older adults, Adaptation, Health promotion

## Abstract

**Background:**

Multi-domain interventions effectively prevent dementia in clinical settings; however, their efficacy within local communities is unclear. This study assesses the feasibility of an adapted multi-domain intervention for dementia prevention in community-dwelling older adults.

**Methods:**

The single-arm trial enrolls 60 participants from two Obu City communities, Japan. Primary outcome: participant retention in the adapted multi-domain intervention; secondary outcomes: health and implementation outcomes. Over 12 months, a team of researchers and public health nurse oversees the study in the first half, gradually shifting the management to public health nurses in the second half. Using the Framework for Reporting Adaptations and Modifications-Enhanced, the clinical programme is adjusted for the local community. It includes a 60-minute exercise and 30-minute group sessions, targeting lifestyle, diet, and social participation.

**Discussion:**

This pioneer study evaluates the feasibility of an adapted intervention programme for dementia prevention in a community setting. Challenges in disseminating dementia prevention programmes warrant further investigation into effective implementation as well as strategies and methods to appeal to the target population. Upon confirming this programme’s feasibility, future studies can further evaluate its broader effectiveness.

**Trial registration:**

The protocol is registered with the Clinical Trials Registry (UMIN-CTR) of the University Hospital’s Medical Information Network, under registration number UMIN000050581.

**Table:** 

Text box 1. Contributions to the literature
• Community-Based Dementia Prevention Feasibility: This study pioneers community-level dementia prevention with insights into adapting multi-domain interventions for older adults.
• Implementation Science Framework: Employing the Framework for Reporting Adaptations and Modifications-Enhanced (FRAME), this study advances the systematic adaptation of evidence-based programs to unique community contexts.
• Barrier Mitigation for Implementation: This study identifies and tackles barriers, including resource constraints and local challenges, enhancing the potential for effective dementia prevention programme delivery.
• Potential Global Impact: By emphasizing feasibility and adherence, this study contributes to the groundwork for extending healthy aging and improving older adults’ quality of life, potentially influencing future global dementia prevention efforts.

## Background

Dementia poses a significant public health challenge impacting affected individuals’ quality of life of affected individuals and imposing a considerable burden on their families and the economy [1]. While there have been reports of a decline in the incidence of dementia in Western countries in recent years [2–4], Japan is experiencing an upward trend [5]. The Hisayama study estimates that Japan will have 650,000–700,000 dementia patients aged 65 and older by 2025, escalating to 800,000–950,000 by 2040, and 850,000–1150,000 by 2060 [5]. Additionally, dementia poses a challenge to achieving the United Nations Sustainable Development Goals (SDGs), particularly Goal 3, which aims to ensure healthy lives and promote well-being for all at all ages. As the global population ages, addressing the rising prevalence of dementia and its associated risk factors becomes essential for achieving this goal. Although the development of disease-modifying drugs targeting Alzheimer’s disease—the most common type of dementia—has progressed with positive results in clinical trials [6], their integration into routine clinical practice requires time. Conversely, non-pharmacological interventions, such as multi-domain intervention programmes for dementia prevention, have demonstrated promising results in suppressing cognitive decline [7]. Given the ease of implementing non-pharmacological therapies in routine clinical and community programmes, research on implementation and dissemination plays a significant role.

An intermediate state between normal cognitive function and dementia, termed mild cognitive impairment (MCI), is potentially reversible [8], making slowing MCI’s progression a preventive measure. In 2017, the Lancet International Commission reported nine modifiable risk factors for dementia: education, midlife hearing loss, hypertension, obesity, smoking in late life, depression, physical inactivity, social isolation, and diabetes [9]. The 2020 report included three additional risk factors: excessive alcohol consumption, head injury, and air pollution—and identified 12 modifiable risk factors that may delay dementia onset by approximately 40% [10]. However, interventions targeting individual dementia risks have limited effects, and in Europe, multi-domain interventions that include exercise, nutrition, and cognitive training are becoming increasingly common [11]. The Finnish Geriatric Intervention Study to Prevent Cognitive Impairment and Disability (FINGER) was a pivotal multicentre randomised controlled trial that applied multi-domain intervention for dementia prevention in 1260 individuals aged ≥ 60 years at risk of developing dementia [7]. The intervention group underwent a two-year period of dietary guidance, exercise instruction, cognitive training, and management of vascular risk factors, while the control group received general health advice. The intervention group showed significant improvement in executive function, processing speed, and overall cognitive scores compared to the control group. While the efficacy of such interventions has been demonstrated in specific regions, such as in the Nordic setting, it remains unclear whether this approach is effective in regions with different lifestyles and cultural backgrounds, or whether ethnic or genetic differences may affect the effectiveness of multi-domain interventions, necessitating further data collection. In July 2017, the World-Wide FINGERS (WW-FINGERS) network was established, and multi-domain intervention trials for dementia prevention began in various countries, advancing global dementia prevention efforts [11]. In Japan, the Randomised Controlled Trial of Multi-domain Intervention for Dementia Prevention (J-MINT) within WW-FINGERS occurred from 2019 to November 2022 [12]. The main analysis is currently underway.

In FINGER, resource-intensive interventions, specifically human and financial, proved effective. Similarly, in J-MINT, efficacy in well-resourced settings is anticipated, necessitating studies on effectiveness and implementation under practical conditions. Furthermore, while the efficacy of well-resourced interventions has been demonstrated, more specific efficacy verification is needed owing to barriers to implementation under realistic conditions.

In recent years, several implementation theories, frameworks, and models have emerged within the field of implementation science; however, their application in ageing research remains limited [13, 14]. The adaptation of evidence-based programmes (EBPs) to particular settings and populations has become common practice in implementation science [15–17]; it is essential for maximising the EBPs’ effectiveness [16, 18, 19]. Furthermore, adaptation is crucial for reducing health disparities, and it offers the possibility of delivering EBPs to populations that have not previously had sufficient access to services [20, 21]. Thus, it is necessary to adapt the preventive intervention to a multi-domain intervention programme to develop a low-resource programme that can be implemented in the community. To ensure adherence to EBP adaptation protocols, the Framework for Reporting Adaptations and Modifications-Enhanced (FRAME) emerges as a valuable tool. FRAME aids in characterising adaptation and promoting implementation, scaling up, dissemination, and sustaining the interventions by clarifying the timing, context, and process of intervention modification [17].

### Objectives

This feasibility trial aims to investigate the feasibility of sustained participation in the adapted intervention and collect data for determining the feasibility of proceeding to the subsequent effectiveness study. The primary objective of the trial is to evaluate the proportion of participants continuing engagement in the adapted multi-domain intervention programme. Secondary objectives include (1) evaluating health outcomes, including cognitive function changes, behavioural change indicators, and other lifestyle indicators; (2) evaluating implementation outcomes, such as fidelity, intervention acceptability, appropriateness, and implementation costs; and (3) verifying that the intervention effect of the original J-MINT intervention remains robust or has potentially strengthened compared to the J-MINT control group. Moreover, this study contributes to achieving SDG Goal 3 by exploring the feasibility of a community-based dementia prevention programme, which, if successful, can improve older adults’ health and well-being, reduce the burden of dementia on individuals and their families, and support the promotion of healthy aging in local communities.

## Methods

### Design

**Fig. 1:**
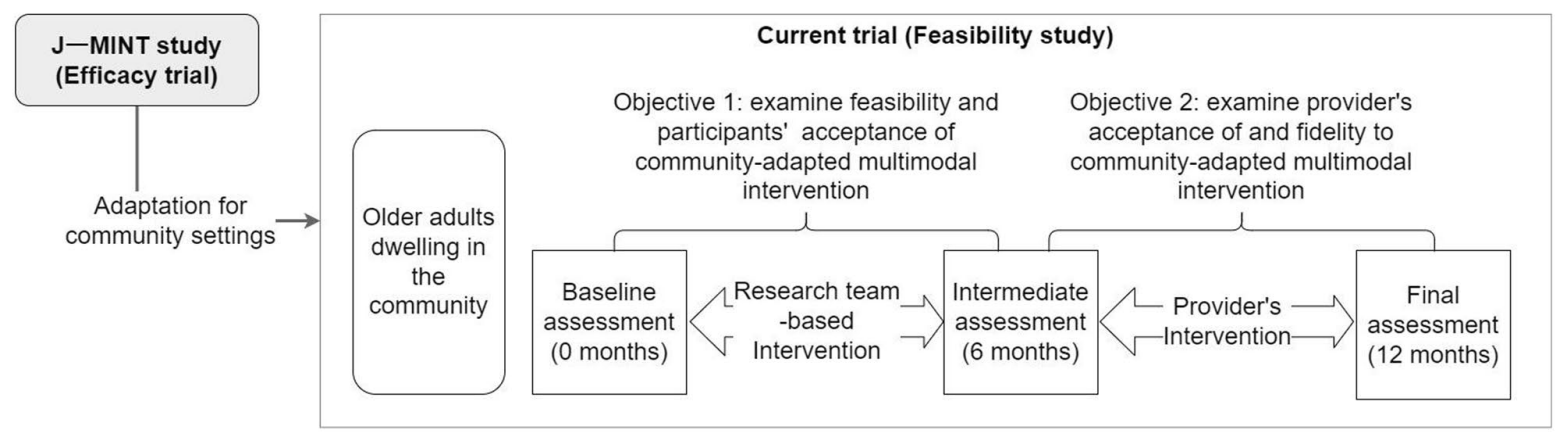
Flowchart of the feasibility study and the evaluation of the adapted dementia prevention programme

The study employs a single-group intervention trial design with a duration of 12 months. In the first half, the researchers (YK, KF, TSu, KU, and TSa), instructors, and local public health nurses will execute the programme as a team (Fig. 1). During the second half, the local public health nurses will take the lead, and outcomes such as compliance with the protocol and fidelity will be evaluated.

### Context and setting

Obu city lies in the Aichi prefecture’s western region, with a population of 90,000 and an ageing segment (aged ≥ 65 years) of 21.4%. While the younger 30–40 age group grows, the ageing proportion, notably those aged ≥ 65, is also rising. Obu estimates approximately 3,152 individuals with dementia among a population of 19,700 aged 65 years or older, with expectations of further increase [22]. Home to the National Centre for Geriatrics and Gerontology, the city is actively addressing dementia-related concerns. Initiatives for a dementia-friendly community include Japan’s first basic ordinance for dementia measures, encompassing diverse actions such as training courses for dementia supporters, preventive health check-ups, dementia cafes, family support programmes, and a monitoring network. The Health Promotion Section of Obu city. ofof supports these efforts, deploying public health nurses, registered dieticians, and social workers.

### Participants

The participants include public health nurses, registered dieticians administering the programme, and the elderly residents receiving it. Specifically, the program engages a full-time public health nurse and a full-time registered dietician from Obu city, with four additional nurses for safety management. These health professionals must hold full-time positions in Obu city, and safety management nurses should possess experience in elderly care prevention programme experience. The study focuses on two communities. Eligible participants include people aged 65–86 years who had not been diagnosed with dementia. Exclusion criteria involve exercise, diet, or water restrictions owing to advanced functional impairment, covering conditions such as bone and joint disorders, renal failure, ischaemic heart disease, and cardiac and pulmonary dysfunction. Further exclusions include dementia diagnosis, a Japanese version of the Montreal Cognitive Assessment (MoCA-J) score ≤ 17 [23], non-Japanese conversational ability, and cognitive function test infeasibility. Participants with a care certification level of ≥ 1 (the LTCI system in Japan provides services based on a seven-level certification [support levels 1–2 and care levels 1–5], determined by disease or functional ability, with higher levels requiring more care) [24] or histories of major depressive disorder, bipolar disorder, schizophrenia, alcohol or drug dependence, a serious illness, or an unstable condition are also excluded. Formal sample size calculation for feasibility evaluation was omitted [25]. The previous J-MINT study, with a continuation proportion of 80%, needed to register 30 cases per community, considering expected dropouts. This adequately meets the sample size for evaluating measured aspects (total of 60), such as participation proportion and lifestyle changes.

### Intervention

#### Adaptation

Engaging researchers from the J-MINT study (original intervention), instructors, supervising project managers, and community public health nurses, interviews were conducted to pinpoint potential barriers to implementing the original intervention within the community. The J-MINT researchers identified a shortage of instructors capable of teaching older adults with MCI. Diverse cognitive and physical dysfunctions in individuals with MCI undermine standardised programmes. Challenges related to implementing the intensive original programme in the community included classroom access limitations and pandemic control operational policies, which added to the challenges. Furthermore, the public health nurses identified shortages of instructors, administrators, and places to provide the programme. In providing physical activity guidance for older individuals with cognitive impairment, specialised knowledge of dementia and appropriate techniques, such as cognitive function training, dual-task exercises, and communication skills, are essential. However, the rarity of specialised instructors for such individuals contributes to the lack of instructors. The unsuitability of intensive interventions within community settings stems from several factors. First, community-based classes often encounter capacity constraints compared to facility-based ones designed to accommodate larger groups. Second, trained instructors’ limited availability and resources restrict of time and effort for classroom instruction and individual guidance.

Considering these barriers, facilitating factors, and the above insights in implementation science, adaptation of the intervention itself becomes essential to develop a low-resource programme suitable for communities. Bash et al. proposed eight adaptation steps (Table 1) [26]. Step 1 involved assessing the needs (health and welfare, planning, and community health promotion) of the management staff and public health nurses in the target area of Obu City, identifying reasons and adaptation goals. In step 2, the authors of the original intervention (TSu and TSa) consulted experts in implementation science (TS and JS) and incorporated their feedback into the study protocol. Step 3 involved three rounds of opinion exchange among public health nurses, nutritionists, and volunteers to discuss barriers, facilitating factors, and solutions.

**Table 1:** Steps of the programme’s adaptation and the status of implementation at the time of study protocol

Step name	Status
1. Conduct a needs assessment	Conducted interviews with J-MINT officials, Obu City department heads (health and welfare, planning, and community development), and public health nurses
2. Consult with experts	The authors of this article developed the original intervention and discussed it with the team, including experts in implementation science.
3. Consult with stakeholders and review assessment data to determine most appropriate and effective EBI	Discussed appropriate EBI to be implemented with frontline public health nurses and community volunteers
4. Decide what needs adapting	Research team and community health nurses Identified items for adaptation within the FRAME
5. Adapt the original EBI	
6. Train staff	Staff and volunteers have been trained
7. Pilot and test the adapted materials	Will be implemented
8. Evaluate	Will be implemented

Abbreviations: J-MINT, The Japan-multimodal intervention trial for prevention of dementia; EBI, Evidence Based Intervention; FRAME, The Framework for Reporting Adaptations and Modifications-Expanded

The method of adaptation was implemented in accordance with the FRAME, under the guidance of implementation science experts, with Steps 4 and 5 conducted by the research team and healthcare professionals [17]. In the adaptation of the original intervention, the core component of the multi-domain intervention, which includes exercise intensity and content, nutritional guidance, cognitive training, and social participation, remains unchanged. However, to provide a sustainable programme in the community, we minimised expensive components. Table 2 outlines adaptation domains, detailing decision-makers, modification specifics, and the rationales behind the decisions and changes. The original intervention provided weekly programmes throughout its one and a half-year intervention period, whereas the community version was shortened to one year to align with the municipal fiscal year. In the target municipality, where exercise programmes were already planned to be provided every two weeks, frequency was doubled. Both versions employed skilled instructors but standardised programme delivery using exercise instruction videos for and health education handbooks. While exercise intensity remained moderate in both versions, the community version monitored symptoms rather than heart rate. For cognitive training, the original version used Brain HQ [27], whereas the community version provided information on a free training app. After the programme was decided, three community healthcare professionals were placed in charge, and 20 staff members received operational training (Step 6). Steps 7 and 8 entail pilot testing and evaluation 8.

**Table 2:** Adaptation from the original version (J-MINT) to the modified (community) program, summarised using the FRAME

Domain/project	Original (J-MINT) programme/provider	Community (Adapted) programme/provider	(1) Who/when decided, (2) What modified, (3) Rationale
No./frequency of interventions	1½ years, weekly (78 sessions)	1 year, every 2 weeks (26 sessions) + exercise classes provided by the local municipality (26 sessions)	(1) At the time of protocol development/municipality and research team collaborated (2) Shortening of period: 1½ years → 1 year (3) To match the municipality’s fiscal year (1 year)
Place of intervention	Dedicated studios in hospitals	Community centre	(1) At the time of protocol development/municipality (2) Change to a location more appropriate to the local context (less space and comfort but better accessibility) (3) To increase the possibility of wide-scale uptake in the region
Exercise instruction methods	Face-to-face guidance + instruction tailored to cognitive and physical functions of subjects/trained instructor	Face-to-face instruction (video + MCI handbook) + instruction tailored to cognitive and physical functions of subjects/trained instructor	(1) During protocol development/municipalities and research team (2) Use of videos and MCI handbook (3) Development of a video and booklet to standardise the J-MINT research experience
Exercise intensity	Moderate (monitored by heart rate)	Moderate (monitored by subjective symptoms)	(1) At the time of protocol development/municipality and research team (2) Change the monitoring method (no change in intensity) (3) To reduce the cost of monitoring and increase the possibility of wide-scale uptake in the community
Nutrition guidance	Nutrition quiz + interviews and telephone support/registered dietitian	Nutrition quiz + MCI handbook/trained instructor	(1) During protocol development/municipality and research team (2) Change to instructor/use of MCI handbook (3) Municipalities already offer individual guidance and telephone support. Additional use of the MCI handbook will provide basic nutritional knowledge and facilitate monitoring in a standardised manner.
Cognitive training	Brain HQ/self-conducted	Introduction to cognitive training + MCI handbook/trained instructor	(1) During protocol development/research team (2) Changed to a more economical intervention (3) To reduce the cost of training and increase the possibility of wide-scale uptake in the community
Lifestyle-related disease management	Regular medical examination/primary care physician	Provision of information by MCI handbook + recommendation for medical check-ups based on medical check-up data/public health nurse	(1) At the time of protocol development/local government + research team (2) Change to municipal initiative/use of handbook (3) Continuity can be ensured if local governments provide recommendations for medical check-ups
Social participation	Self-monitoring	Monitoring + group work/implemented by self and shared with the group	(1) During protocol development/research team (2) Additional group work (3) Sharing among participants fosters interaction and sustainability, which in turn reinforces behavioural change.
Provision of health information	Providing information in brochures/research staff	Providing information using MCI handbook/trained instructor	(1) During protocol development/research team (2) Use of MCI handbook (3) MCI handbook provides comprehensive knowledge of dementia prevention

Abbreviations. J-MINT, The Japan-multimodal intervention trial for prevention of dementia; MCI, Mild Cognitive Impairment

#### Programme structure

During the first six months, the research team will lead the community intervention, followed by local public health nurses for the subsequent six months. The research programme will be conducted once every two weeks (26 times a year), with the local government-implemented exercise programme every other week, resulting in weekly engagement. The research programme will consist of 60 min of exercise and 30 min of group work. The group work will feature content related to dementia prevention (e.g. lifestyle diseases, dietary habits, social participation), and facilitators from the research team and the local government will promote interaction among participants. The specific content of the intervention is explained below.

### Lifestyle-related Disease management

#### Exercise instruction

An instructor with experience in teaching people with MCI will guide the group exercise. The exercise programme comprises 25 min of stretching, strength training, and balance training; 20 min of aerobic exercise; and 15 min of dual-task training that combines physical exercise with cognitive tasks [28]. In aerobic exercise, participants will monitor their symptoms under a moderate-intensity load. To ensure safe exercise, the public health nurse will first conduct a medical interview and measure blood pressure and heart rate. The exercise intervention will not be implemented if any of the following criteria are met: systolic blood pressure of ≥ 180 mm Hg or a diastolic blood pressure of ≥ 110 mm Hg at rest, heart rate of ≥ 110 bpm or ≤ 50 bpm at rest, any irregular heartbeat, or worsening of chronic symptoms (such as joint pain).

To motivate participants to improve their physical activity levels, self-monitoring using an activity record sheet (life note) will be implemented. On the sheet, participants will record their daily steps, whether they have exercised or not, and the exercises completed after setting goals. Additionally, participants will record daily information about their diet and weight, as well as other lifestyle-related information. The sheet will be checked each week, and feedback will be given on effective exercise methods, ways to increase physical activity, and specific activity goals.

#### Nutrition guidance programme

The instructor will provide nutritional information using the *MCI Handbook* and recommend self-monitoring through food diaries. The *MCI Handbook* has questions such as ‘Can MCI be suppressed through diet?’, ‘What types of food can suppress the progression of MCI?’, and ‘Can MCI be suppressed through the use of supplements or nutritional supplements?’. During individual guidance, participants will receive direction on nutrient components, eating behaviours, and diversity of food intake. Appropriate dietary intake amounts will be determined from the *Japanese Dietary Reference Intakes for Elderly 2020*, which presents information about the standard intake amounts and nutritional balance of protein, lipids, carbohydrates, vitamins, minerals, and energy for people aged 65–74 and ≥ 75 years. Recognising the diet’s association with improved physical and cognitive performance in older people in Japan [29, 30], participants will be advised to improve their diet’s diversity and balance.

#### Cognitive training and social participation

Effective dementia-preventive cognitive training programmes, including the Kayoinoba smartphone app developed by the National Center for Geriatrics and Gerontology [31], will be introduced. Participants will be encouraged to use these programmes for 20 min a day, at least thrice a week. Those who do not have smartphones will be encouraged to use the *MCI Handbook* and cognitive training in book form.

To encourage social participation, instructors will provide information from the *MCI Handbook*, with the goal of encouraging at least three outings a week. During a 15-minute group work session after each class, participants will be asked to report on their physical activities and social participation levels during the week.

### Assessment

Following participant consent, baseline observations and examinations will be conducted. Specific observations and examinations will be carried out after six (± 2) months of participation and at the 12-month mark. The items and participants for each of the health and implementation outcomes are presented in Table 3. Each measure is described in detail.

**Table 3:** Outcome measures, informants, and timeframe of assessments

Construct	Measure/indicator	Informant		Timeframe (months)		
		Participants	Providers	0	6	12
Cognitive function	MoCA-J	×		×	×	×
Physical function/status	Gait speed	×		×	×	×
	Grip strength	×		×	×	×
	Body mass index	×		×		×
Awareness of healthy activities	Behavioural modification stages	×		×	×	×
Comprehensive geriatric assessment	Lifestyle	×		×	×	×
	Variation in diet	×		×	×	×
	Social participation	×		×	×	×
	Health-related quality of life	×		×	×	×
	Physical activities	×		×	×	×
Implementation outcomes	Feasibility	×			×	
	Fidelity		×			×
	Participants’ adherence	×			×	
	Acceptability	×	×		×^a^	×^b^
	Appropriateness		×			×
	Cost			×*	×*	×*

*Gather information prospectively; ^a^ obtain from participants; ^b^ obtain from providers

Abbreviations. MoCA-J: The Japanese version of Montreal Cognitive Assessment

#### Screening survey

The screening survey will utilise the MoCA-J tool, employed for dementia screening, MCI, assessment, and evaluation of visuospatial and executive functions, naming, memory, attention, recitation, word recall, abstract concepts, delayed replay, and disorientation [32, 33]. The tool is considered reliable and has been previously validated [23]; patients who score < 26 on the MoCA-J are more likely to be diagnosed with MCI. A previous study tested the feasibility of administering the MoCA-J in web format and reported high reliability and satisfaction levels [34]. This study will conduct neuropsychological testing in web format as required.

#### Survey items related to health behaviours

The status of health behaviours related to dementia risk will be assessed by a survey. For each regular exercise (at least thrice a week), dietary modification, social participation, and cognitive training, five behavioural change stages will be assessed using the transtheoretical model [35]. Regarding regular exercise, the options include ‘I do not exercise regularly, and do not intend to exercise in the future (within the next 6 months)’ (precontemplation phase); ‘I do not exercise; however, I intend to start exercising in the future (within the next six months)’ (contemplation); ‘I intend to start exercising now (within a month), and I am preparing for it, or I am exercising regularly (at least three times a week) but not regularly’ (preparation); ‘I have started regular (at least 3 times a week) exercise within the last six months’ (action); and ‘I have been exercising regularly (at least three times a week) for more than six months’ (maintenance).

#### Comprehensive functional assessment

##### (1) Lifestyle

A questionnaire will be used to evaluate items related to dementia risk: exercise habits, sleep, medication and medical history, cognitive activities, social activities, subjective cognitive impairment, hobbies and activities, friendships, eating habits, and disease information.

##### (2) Food intake diversity

The Food Diversity Score, consisting of 11 items, has been adapted [36] to assess participants’ frequency of consuming 13 food items over the past week, including cereals, fish and shellfish, meat, eggs, milk, dairy products, beans, seaweed, potatoes, fruit, and nuts. Each item is scored as 1 (almost every day), 0.5 (once every 2 days), 0.25 (once or twice a week), and 0 (rarely eaten).

##### (3) Social participation

Social engagement evaluation will involve a questionnaire examining participation across eight types of groups [37]: local community, including neighbourhood associations, senior citizen clubs, and fire-fighting teams; hobby groups; sports groups or clubs; politics-related organisations or groups; industry-related or trade associations; religion-related organisations or groups; volunteer groups; and other groups.

##### (4) Frailty-related index

The Simple Frailty Index [38] will be used to evaluate the degree of frailty among older adults. The degree of frailty is assessed using five items (weight loss, walking speed, movement, memory, and fatigue); one point is given for each item that applies. A score of ≥ 3 is considered frail, and a score of 1–2 is considered pre-frail.

##### (5) Health-related quality of life

To evaluate the health-related quality of life, a health index of the EQ-5D will be used. It comprises five dimensions: mobility, self-care, pain/​discomfort, usual activities, and anxiety/depression. The scores on these dimensions are summed, and a single index score (utility) is calculated with reference to previous studies [39].

##### (6) Physical performance

Physical performance will be assessed by measuring the usual gait speed over a distance of 2.4 m on a walkway [40]. Hand grip strength will be measured for both hands using a standard digital hand grip dynamometer (Takei Scientific Instruments Co., Ltd, Japan,) while participants stand with their shoulders adducted and neutrally rotated and the elbows fully extended [41].

### Implementation outcomes

The study will measure implementation outcomes, including participant adherence, fidelity, feasibility, acceptability, appropriateness, and cost—for both programme providers and programme participants [42]. Quantitative and qualitative methods will be used to collect data, depending on the purpose. The primary endpoint is the participant’s adherence at 6 months—the proportion of participants who continue to participate in the adapted multi-domain intervention programme. Fidelity is measured as the degree to which procedures and regulations are followed in the programme implementation. Based on a predesigned checklist of procedures, a score of 100 is given if the intervention provider fulfils all procedures. Additional implementation outcomes will be measured qualitatively using semi-structured interviews. Feasibility will be measured for the implementers at 6 and 12 months to assess whether multi-domain interventions are feasible (e.g. ‘Do the multi-domain interventions seem to be implementable?’). The acceptability of the interventions will be measured for the implementers and participants at 6 and 12 months to assess whether they agree with, prefer, or are satisfied with the programme content (e.g. ‘Do the multi-domain interventions seem appealing to you?’). The appropriateness of the intervention will be measured for implementers at 12 months and participants at 6 and 12 months, to assess whether the programme is appropriate for the site (e.g. ‘Do the multi-domain interventions seem fitting?’). Each of these interview items is based on validated measurements [43]. All interviews will be conducted by researchers trained in qualitative research, using a combination of individual interviews and focus groups. While community-dwelling older adults will participate in qualitative research, not all will undergo individual interviews. These individual interviews will be specifically aimed at gaining deeper insights into qualitative implementation outcomes, especially acceptability and appropriateness. For example, in situations of low acceptability, individual interviews will be particularly valuable. Programme providers will participate in focus group interviews to gain perspectives from group dynamics and interactions. Implementation costs will be determined by prospectively collecting information on time spent by members of the research team from programme development to implementation; costs of supplies and equipment will also be determined.

### Analysis

The primary outcome measures of all participants will be analysed. The proportion of individuals sustaining programme engagement will be calculated from the initial assessment to the six-month follow-up for those attending ≥ 60% of classes (with at least half of the classes attended). The rationale for setting the proportion is based on a study that examined the intervention effects by participants’ adherence level in the FINGER study [7]. The above-mentioned study found that cognitive decline was suppressed when at least 50% of individuals participated and at least 57% of the group attended classes.

For the secondary outcome measures, stratification will be performed for older adults with a risk of dementia (MCI) and those without. The programme adaptation will be evaluated to determine whether the effect of the original intervention has weakened or been maintained compared to the control group in the original study. Specifically, changes before and after the intervention will be analysed for health behaviour and comprehensive functional evaluations. For continuous variables, summary statistics will be calculated for changes in the six-month period from the initial assessment and tested using paired sample t-tests or, for non-parametric data, Wilcoxon’s signed-rank tests. For categorical variables, the frequency and proportion of cases corresponding to each category and 95% confidence interval will be calculated at each time point. The confidence interval will be calculated using the Clopper–Pearson method, which provides an accurate estimation for binomial proportion confidence intervals and is particularly useful when dealing with small sample sizes or extreme proportions [44]. If necessary, changes before and after the intervention will be compared using McNemar’s test, a statistical test used to analyse paired nominal data. It is appropriate for our design as it evaluates changes in two related groups over time [45]. Qualitative data used for implementation outcomes will be analysed by summary content analysis [46, 47]. Specifically, recorded interview data will be transcribed verbatim. Important concepts and themes will be coded for category creation. Coding will be performed by a researcher confirmed by another. The research team will validate categories aggregating similar codes. Qualitative analysis will benefit from consultation with a qualitative research expert.

### Ethical consideration

Study procedures have been reviewed and approved by the Institutional Review Board. The protocol is registered with the Clinical Trials Registry (UMIN-CTR) of the University Hospital’s Medical Information Network, under registration number UMIN000050581. Participants will be fully informed about the purpose, nature, and potential risks associated with participation and will be required to provide written informed consent before enrolling in the study.

## Discussion

An ongoing international clinical trial is investigating a multi-domain intervention for dementia prevention. However, this study’s focus on community-specific adaptation and feasibility is a pioneering endeavour. Previous studies have raised concerns about participants’ adherence to high-intensity intervention programmes and the lack of beneficial effects because of low adherence. Confirming the feasibility of the adapted dementia prevention programme in the present study can pave the way for its broad implementation in Japanese community settings. Moreover, using FRAME to describe the systematic process of applying the adaptation procedure will enhance our understanding of how adaptation is related to outcomes.

The Japanese Ministry of Health, Labour, and Welfare recommends promoting health classes and risk assessment checks for dementia among older adults as part of their dementia prevention programme. While dementia prevention programmes have been implemented in municipalities and care facilities, EBPs have not yet been widely adopted. Challenges such as the training of program providers and development of evaluation indicators are cited as hindrances to dissemination [48]. Internationally, there are complex issues such as low recognition and knowledge of dementia, insufficient research on the effectiveness and implementation of programmes, and cultural and access-related barriers [49, 50]. As specialised knowledge and expertise are required to implement these programmes, the interviews with this study’s intervention providers reveal that appropriate facilities and professionals are limited. Moreover, participation in such programmes may be perceived as a significant barrier owing to the associated costs and time constraints, and prejudice against individuals with dementia may reduce interest in prevention programmes [51, 52].

A limitation of this study is its focus on Obu city alone, which may limit the direct applicability of the findings to other regions or settings. Given that the city has actively promoted dementia policies, the residents might possess a higher level of dementia literacy, which could influence the study outcomes. Furthermore, the on-site nature of the programme restricts participation to those with maintained physical functions. Moreover, the participant pool mainly comprises those already involved in existing programmes in Obu city. While this ensures a certain number of participants, the potential barriers to recruiting new participants for similar programs in the future might not be fully understood. This highlights the need for accessible delivery methods, such as online platforms, for broader reach. However, despite these limitations, this study boasts strengths. Concentrating on a specific community and employing implementation science methodologies tailors methods to specific contexts, offering insight into real-world intervention challenges. Addressing both the feasibility and challenges, this study lays the foundation for broader dementia prevention program implementation.

Multiple factors can hinder the dissemination of evidence-based dementia prevention programmes in local communities. It is necessary to develop implementation strategies and consider ways to effectively reach people for future implementation strategies. After confirming the feasibility of the intervention, we will develop an implementation strategy and conduct an Effectiveness–Implementation Hybrid Design Study by combining evidence-based interventions and implementation strategies.

Should the intervention effectively cater to community-dwelling older adults, it holds potential to become a standard approach to dementia prevention. Furthermore, the results will contribute to extending the healthy life expectancy of older adults and improving their health-related quality of life. The results can be used as a basis for formulating dementia policies and contributing to preventive care and medical economics.

## Data Availability

An anonymous analysed data will be available to researchers upon reasonable request to the corresponding author.

## List of abbreviations

EBPs: Evidence-based programmes
FRAME: Framework for Reporting Adaptations and Modifications-Enhanced
MCI: Mild cognitive impairment
